# Usage of compromised lung volume in monitoring steroid therapy on severe COVID-19

**DOI:** 10.1186/s12931-022-02025-6

**Published:** 2022-04-29

**Authors:** Ying Su, Ze-song Qiu, Jun Chen, Min-jie Ju, Guo-guang Ma, Jin-wei He, Shen-ji Yu, Kai Liu, Fleming Y. M. Lure, Guo-wei Tu, Yu-yao Zhang, Zhe Luo

**Affiliations:** 1 Department of Critical Care Medicine, Zhongshan Hospital, Fudan University, Shanghai, China; 2 School of Information Science and Technology, ShanghaiTech University, Shanghai, China; 3 Department of Radiology, Renmin Hospital of Wuhan University, Wuhan, China; 4 MS Technologies, Rockville, MD USA; 5 Department of Critical Care Medicine, Xiamen Branch, Zhongshan Hospital, Fudan University, Xiamen, China; 6 Shanghai Key Lab of Lung Inflammation and Injury, Shanghai, China

**Keywords:** Steroid, Quantitative computed tomography, Compromised lung volume, COVID-19

## Abstract

**Background:**

Quantitative computed tomography (QCT) analysis may serve as a tool for assessing the severity of coronavirus disease 2019 (COVID-19) and for monitoring its progress. The present study aimed to assess the association between steroid therapy and quantitative CT parameters in a longitudinal cohort with COVID-19.

**Methods:**

Between February 7 and February 17, 2020, 72 patients with severe COVID-19 were retrospectively enrolled. All 300 chest CT scans from these patients were collected and classified into five stages according to the interval between hospital admission and follow-up CT scans: Stage 1 (at admission); Stage 2 (3–7 days); Stage 3 (8–14 days); Stage 4 (15–21 days); and Stage 5 (22–31 days). QCT was performed using a threshold-based quantitative analysis to segment the lung according to different Hounsfield unit (HU) intervals. The primary outcomes were changes in percentage of compromised lung volume (%CL, − 500 to 100 HU) at different stages. Multivariate Generalized Estimating Equations were performed after adjusting for potential confounders.

**Results:**

Of 72 patients, 31 patients (43.1%) received steroid therapy. Steroid therapy was associated with a decrease in %CL (− 3.27% [95% CI, − 5.86 to − 0.68, *P* = 0.01]) after adjusting for duration and baseline %CL. Associations between steroid therapy and changes in %CL varied between different stages or baseline %CL (all interactions, *P* < 0.01). Steroid therapy was associated with decrease in %CL after stage 3 (all *P* < 0.05), but not at stage 2. Similarly, steroid therapy was associated with a more significant decrease in %CL in the high CL group (*P* < 0.05), but not in the low CL group.

**Conclusions:**

Steroid administration was independently associated with a decrease in %CL, with interaction by duration or disease severity in a longitudinal cohort. The quantitative CT parameters, particularly compromised lung volume, may provide a useful tool to monitor COVID-19 progression during the treatment process.

*Trial registration* Clinicaltrials.gov, NCT04953247. Registered July 7, 2021, https://clinicaltrials.gov/ct2/show/NCT04953247

## Background

Currently, the coronavirus disease 2019 (COVID-19) pandemic caused by severe acute respiratory syndrome coronavirus-2 (SARS-CoV-2) has spread across the world [1]. As of April 1, 2022, 486,761,597 people have been diagnosed with COVID-19, including 6,142,735 deaths, according to the international World Health Organization (WHO) dashboard. Among various therapies for COVID-19, corticosteroid therapy has been proven to be effective for critical cases. The RECOVERY trial first reported the effectiveness of corticosteroid therapy in reducing mortality in patients with COVID-19 receiving oxygen supplementation or invasive mechanical ventilation [2]. The WHO REACT meta-analysis that included data from seven randomized clinical trials (RCTs) also demonstrated that the use of systemic corticosteroids was associated with lower 28-day all-cause mortality in critically ill patients with COVID-19 [3]. Although corticosteroid therapy represents a milestone in the management of COVID-19, many questions remain unanswered [4]. The optimal type of corticosteroids, timing of initiation, dose, mode of administration, duration, and dose tapering are still unclear. An approach to resolve these issues is to develop accurate tools to assess or monitor the progression of COVID-19 during the corticosteroid therapy process.

Chest computed tomography (CT) plays an important role in screening, diagnosing, and evaluating longitudinally patients with COVID-19 [5, 6]. Visual assessment of pulmonary lesions on chest CT scans has been proven to be valuable in predicting outcome and assessing progression [7, 8]. However, inter- or intra-observer variation in traditional visual assessment of chest CT images increases the challenge of therapeutic assessment [9, 10]. Quantitative computed tomography (QCT) analyses have recently been widely used to evaluate various pulmonary diseases [11]. QCT enable to extract quantitative data from medical images and increases the reproducibility of evaluation, thus, serving as a potential tool for monitoring disease progression and treatment response [12]. This may be particularly valuable in circumstance of minor effect as new therapies are developed and evaluated [7].

We hypothesize that QCT can serve as a tool for monitoring steroid treatment response during the course of disease. Our preliminary studies have confirmed the value of QCT in monitoring the progression and clinical decision-making in patients with COVID-19 [13, 14]. However, the effect of steroids on quantitative chest CT parameters during the treatment process remains unknown. In this retrospectively study, we aimed to assess the association between steroid administration and QCT variables in a longitudinal cohort with COVID-19.

## Methods

### Study design and participants

The present study was approved by the Ethics Committee of Renmin Hospital of Wuhan University (WDRY2020-K048) and was performed in accordance with the Declaration of Helsinki. Written informed consent was waived by the Ethics Committee in the setting of COVID-19 crisis in Wuhan.

From February 7, 2020 to February 17, 2020, consecutive patients with confirmed COVID-19 admitted to the east campus of Renmin Hospital of Wuhan University were screened. The diagnosis of COVID-19 was based on the detection of SARS-CoV-2 nucleic acid by a real-time RT-PCR assay.

The inclusion criteria were as follows: (1) age ≥ 18 years and (2) patients with severe or critical COVID-19. Exclusion criteria included (1) hematological or solid malignancies, (2) patients with less than two CT scans during hospital stay, and (3) systemic corticosteroid or immunosuppressive therapy in the previous 6 weeks. The severity of COVID-19 was defined according to WHO interim guidance or the sixth edition of the Chinese national guidelines on the diagnosis and treatment for COVID-19 [15, 16]. Patients were considered to have a severe infection if they met any of the following conditions: respiratory distress and a respiratory rate of > 30 times/min; oxygen saturation on room air at rest < 93%; and partial arterial oxygen pressure (PaO_2_)/fraction of inspiration oxygen (FiO_2_) ≤ 300 mmHg. Patients were considered to be in a critical state if they met any of the following conditions: respiratory failure requiring mechanical ventilation, shock, and dysfunction of other organs requiring ICU management.

### Data collection

Data regarding baseline demographic features, co-morbidities, and clinical characteristics on hospital arrival, including symptoms, vital signs, and interventions during hospital stay, were retrospectively collected by two trained reviewers.

Because there was no consensus on the use of steroids in the early stage of the COVID-19 pandemic, all steroid therapies were initiated at the time of admission at the discretion of attending physicians on the basis of clinical symptoms and CT images. According to our previous experience [17–21], intravenous methylprednisolone at a dose of 0.5–1.0 mg/kg every 12 h was initiated for 5 days or until oxygen saturation improved, followed by gradual tapering by 0.5 mg/kg every 3–5 days.

Other therapeutic interventions such as the use of antibiotics, ventilation, laboratory testing, and hemodynamic management were performed following the sixth edition of the Guidelines on the Diagnosis and Treatment of COVID-19 published by the National Health Commission of China.

### CT protocol

All patients underwent CT examination at hospital admission. Serial chest CT scans were performed during hospital stay. All CT scans were performed within a single inspiratory phase on a commercial multidetector CT scanner (GE Optima CT680). To minimize motion artifacts, CT images were acquired during a single breath-hold. Standard lung algorithm settings were used as follows: 120 kV and automatic tube current (180–400 mA); iterative reconstruction technique; detector, 64 mm; rotation time, 0.35 s; section thickness, 5 mm; collimation, 0.625 mm; pitch, 1.5; matrix, 512 × 512. Based on the time interval between hospital admission and CT scan, we designated five stages in this study: stage 1 (baseline CT scans, in which CT scans were acquired at hospital admission); stage 2 (CT scans acquired > 3 to 7 days after admission); stage 3 (CT scans acquired > 8 to 14 days after admission); stage 4 (CT scans acquired > 15 to 21 days after admission); and stage 5 (CT scans acquired > 22 to 31 days after admission).

### CT image analysis

For patient lung parenchyma segmentation, we performed a volumetric analysis in 3D Slicer (http://www.slicer.org) via the Lung CT Analyzer project (https://github.com/rbumm/SlicerLungCTAnalyzer/) [22]. We extracted right and left lung segments from individual CT slices. For unsatisfactory lung segmentation, we refined the lung contours with the manual segmentation tool implemented in ITK-SNAP (www.itksnap.org) [23]. The trachea was excluded from the lung segmentation, while segmental arteries and bronchi were included. We then further divided the lung region into four components and computed the volume of each lung component, which were considered as the percentages of the total volume. According to different Hounsfield unit (HU) intervals in the quantitative chest CT scan, we divided each lung into nonaerated lung volume (%NNL, 100 to − 100 HU), poorly aerated lung volume (%PAL, − 101 to − 500 HU), normally aerated lung volume (%NAL, − 501 to − 900 HU), and hyperinflated lung volume (%HI, − 901 to − 1000 HU) regions [24, 25]. The additional “compromised lung” volume (%CL) was considered as the sum of %PAL and %NNL (− 500 to 100 HU). The authors (Ze-song Qiu, Jin-wei He, and Yu-yao Zhang) who performed quantitative CT analysis were blinded to clinical characteristics and outcomes.

### Outcome assessment

To monitor COVID-19 progression during the treatment process, we chose changes in the percentage of compromised lung volume (Δ%CL) at different stages (Δ%CL = %CL at different stages-baseline %CL) as the primary outcome. The negative value of Δ%CL thus reflected clinical improvement.

The secondary outcomes were changes in the percentage of NNL (Δ%NNL = NNL at different stages-baseline NNL), PAL (Δ%PAL = %PAL at different stages-baseline %PAL), NAL (Δ%NAL = %NAL at different stages-baseline %NAL), and HI (Δ%HI = %HI at different stages-baseline %HI) at different stages. Under these circumstances, clinical improvement was reflected by the negative value of Δ%NNL and Δ%PAL, and the positive value of Δ%NAL.

### Statistical analysis

Categorical variables were expressed as numbers and percentages. The normality of the distribution for continuous variables was assessed by the Kolmogorov–Smirnov test. Continuous variables were expressed as the mean and standard deviation (SD) or median and interquartile range [IQR]. The chi-square test or Fisher’s exact test was used for categorical variables, while the Student’s t test or Mann–Whitney U test was used for continuous variables, as appropriate. For serial quantitative CT parameters, we tested differences between two groups over time by using repeated-measures analysis of variance (ANOVA) with no imputation for missing values. The Šidák multiple comparisons correction was used to compare each stage against the baseline (Stage 1).

To longitudinally assess the association between steroid administration and changes in the percentage of quantitative CT parameters, generalized estimating equations (GEE) were used so that correlations between repeat CT quantitative parameters for an individual patient could be considered. The outcome variables Δ%CL, Δ%NNL, Δ%PAL, Δ%NAL, and Δ%HI were set as time-dependent variables because they were measured at each examination visit. Associations between steroid use and Δ%CL, Δ%NNL, Δ%PAL, Δ%NAL, and Δ%HI at different stages were assessed using GEE with correlations between repeated measures on participants modeled using an exchangeable structure to implement linear regression models. Covariates included in the GEE models were first screened by univariate GEE analysis, and those covariates with *P* < 0.10 were introduced in multivariate GEE models. These models were finally adjusted for duration and baseline quantitative CT parameters. Interactions between steroid use and duration (steroid use*duration) or between steroid use and baseline quantitative CT parameters (steroid use*baseline quantitative CT parameters) were tested by multivariate GEE analysis. Subsequent analyses assessed the associations between steroid use and changes in the percentage of quantitative CT parameters at different stages by linear regression models. We also assessed the associations between steroid use and changes in the percentage of quantitative CT parameters in relation to different baseline quantitative CT parameters. Patients were classified into high (**≥ **median level) and low groups (< median level) according to baseline quantitative CT parameters. The β coefficients and 95% confidence intervals (CI) were calculated. All statistical analyses were performed with SPSS software package, version 15.0 (SPSS Inc., Chicago, IL, USA), and two-sided *P* values of less than 0.05 were considered to be statistically significant.

## Results

### Patient characteristics

**Fig. 1:**
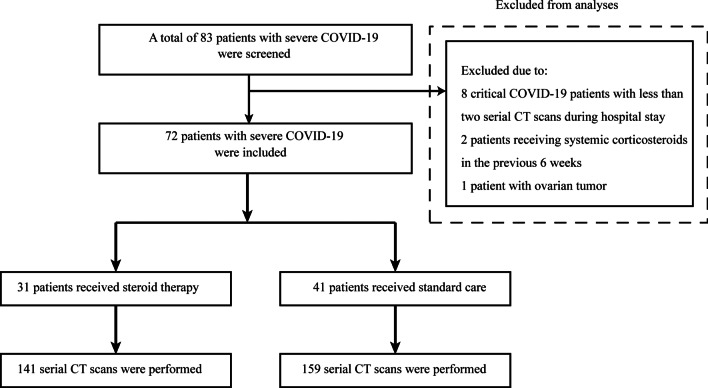
The enrollment of patients. COVID-19, coronavirus disease 2019; CT, computed tomography

Between February 7 and February 17, 2020, a total of 83 patients with severe or critical COVID-19 were screened for inclusion. Of these patients, 11 patients were excluded, including eight critical COVID-19 patients with less than two serial CT scans during hospital stay, two patients receiving systemic corticosteroids or immunosuppressive therapy in the previous 6 weeks, and one patient with solid malignancy. Within the study period, 72 patients with severe COVID-19 were finally included in this study (Fig. 1).

Table 1 presents the baseline characteristics of patients with severe COVID-19. The median age of the 72 patients was 63 years [IQR 49 to 69 years], and 51.4% of the patients were male. Fever was the most common symptom (90.3%), followed by fatigue (87.5%), cough (73.6%), and dyspnea (55.6%). Thirty-one patients (43.1%) received steroid therapy. Steroid therapy was started at a median of 14 [11, 19] days from onset of symptom. The median duration of steroid therapy was 13 [7, 18] days. The baseline characteristics between patients receiving steroid therapy and those not receiving steroid therapy were similar (Table 1). The vital signs and the PaO_2_/FiO_2_ ratio on admission were also similar between the two groups.

**Table 1:** Clinical characteristics of patients with severe COVID-19

	Entire cohort	Steroid group	Standard care group	*P* value
Number of patients	72	31	41	
Age (years)	63 [49,69]	65 [55,72]	62 [48,69]	0.31
Gender (male), n (%)	37 (51.4)	14 (45.2)	23 (56.1)	0.48
Smoking history, n (%)	9 (12.5)	4 (12.9)	5 (12.2)	1.00
Comorbidities				
Hypertension, n (%)	22 (30.6)	11 (35.5)	11 (26.8)	0.45
Diabetes mellitus, n (%)	16 (22.2)	7 (22.6)	9 (22.0)	1.00
CAD, n (%)	4 (5.6)	2 (6.5)	2 (4.9)	1.00
COPD, n (%)	1 (1.4)	0 (0)	1 (2.4)	1.00
Cerebrovascular disease, n (%)	1 (1.4)	1 (3.2)	0 (0)	1.00
Chronic renal disease, n (%)	2 (2.8)	1 (3.2)	1 (2.4)	1.00
Signs and symptoms				
Fever, n (%)	65 (90.3)	30 (96.8)	35 (85.4)	0.23
Cough, n (%)	53 (73.6)	24 (77.4)	29 (70.7)	0.60
Sputum production, n (%)	10 (13.9)	4 (12.9)	6 (14.6)	1.00
Fatigue, n (%)	63 (87.5)	28 (90.3)	35 (85.4)	0.72
Headache, n (%)	4 (5.6)	1 (3.2)	3 (7.3)	0.63
Dyspnea, n (%)	40 (55.6)	20 (64.5)	20 (48.8)	0.23
Nausea or vomiting, n (%)	13 (18.1)	6 (19.4)	7 (17.1)	1.00
Diarrhea, n (%)	15 (20.8)	7 (22.6)	8 (19.5)	0.77
Anorexia, n (%)	4 (5.6)	3 (9.7)	1 (2.4)	0.31
Myalgia or arthralgia, n (%)	7 (9.7)	4 (12.9)	3 (7.3)	0.45
Onset of symptom to first CT scan (days)	14 [11,17]	14 [11,19]	13 [11,16]	0.16
Vital signs at hospital admission				
Altered mental status, n (%)	2 (2.8)	2 (6.5)	0 (0)	0.18
Heart rate (beats/minute)	88 [78,102]	85 [77,105]	90 [81,100]	0.83
Respiratory rate (breaths/minute)	24 [23,31]	25 [24,32]	24 [23,29]	0.20
Systolic blood pressure (mm Hg)	132 [122,145]	132 [123,147]	131 [120,144]	0.44
Diastolic blood pressure (mm Hg)	78 [71,83]	79 [72,83]	76 [70,84]	0.54
Respiratory status assessment				
PaO_2_ on admission (mmHg)	67 [61,86]	66 [59,84]	68 [63,87]	0.49
PaCO_2_ on admission (mmHg)	39 [34,42]	39 [33,41]	40 [36,43]	0.36
PaO_2_/FiO_2_ on admission (mmHg)	256 [226,277]	246 [195,279]	258 [228,277]	0.40
Respiratory support				
High flow nasal oxygen, n (%)	14 (19.4)	11 (35.5)	3 (7.3)	< 0.01
Non-invasive mechanical ventilation, n (%)	2 (2.8)	2 (6.5)	0 (0)	0.18
Invasive mechanical ventilation, n (%)	1 (1.4)	1 (3.2)	0 (0)	0.43
Renal replacement therapy, n (%)	1 (1.4)	0 (0)	1 (2.4)	1.00
Hospital mortality, n (%)	0 (0)	0 (0)	0 (0)	-
Duration of viral shedding after COVID-19 onset (days)	25 [18,31]	27 [22,34]	23 [17,29]	0.03
Hospital length of stay (days)	33 [27,39]	36 [31,42]	29 [24,36]	< 0.01

Data are expressed as the median with interquartile range (IQR) in square brackets for non-normally distributed data. Continuous variables are shown as the mean ± SD or median [IQR], as appropriate. Categorical variables are shown as number (%)

COVID-19, coronavirus disease 2019; CAD, coronary artery disease; COPD, chronic obstructive pulmonary disease; FiO_2_, fraction of inspired oxygen; PaO_2_, partial pressure of oxygen

During hospital stay, more patients in the steroid therapy group were treated with high-flow nasal oxygen than standard care group (35.5% vs. 7.3%, *P* < 0.01). However, the proportion of invasive or noninvasive ventilation between the two groups was comparable. All patients survived to discharge from hospital in this study. The length of hospital stay was longer in patients receiving steroid therapy than in those not receiving steroid therapy (36 vs. 29 days, *P* < 0.01).

### Quantitative CT parameters over time in the steroid therapy group and the standard care group

All CT scans were classified into five stages according to the interval between hospital admission and follow-up CT scans: Stage 1 (T1, at admission); Stage 2 (T2, 3–7 days); Stage 3 (T3, 8–14 days); Stage 4 (T4, 15–21 days); and Stage 5 (T5, 22–31 days). Three hundred chest CT scans were longitudinally collected from 72 patients with severe COVID-19. Among them, 72 CT scans were collected at Stage 1, 62 CT scans at Stage 2, 65 CT scans at Stage3, 56 CT scans at Stage 4 and 45 CT scans at Stage 5.

**Fig. 2:**
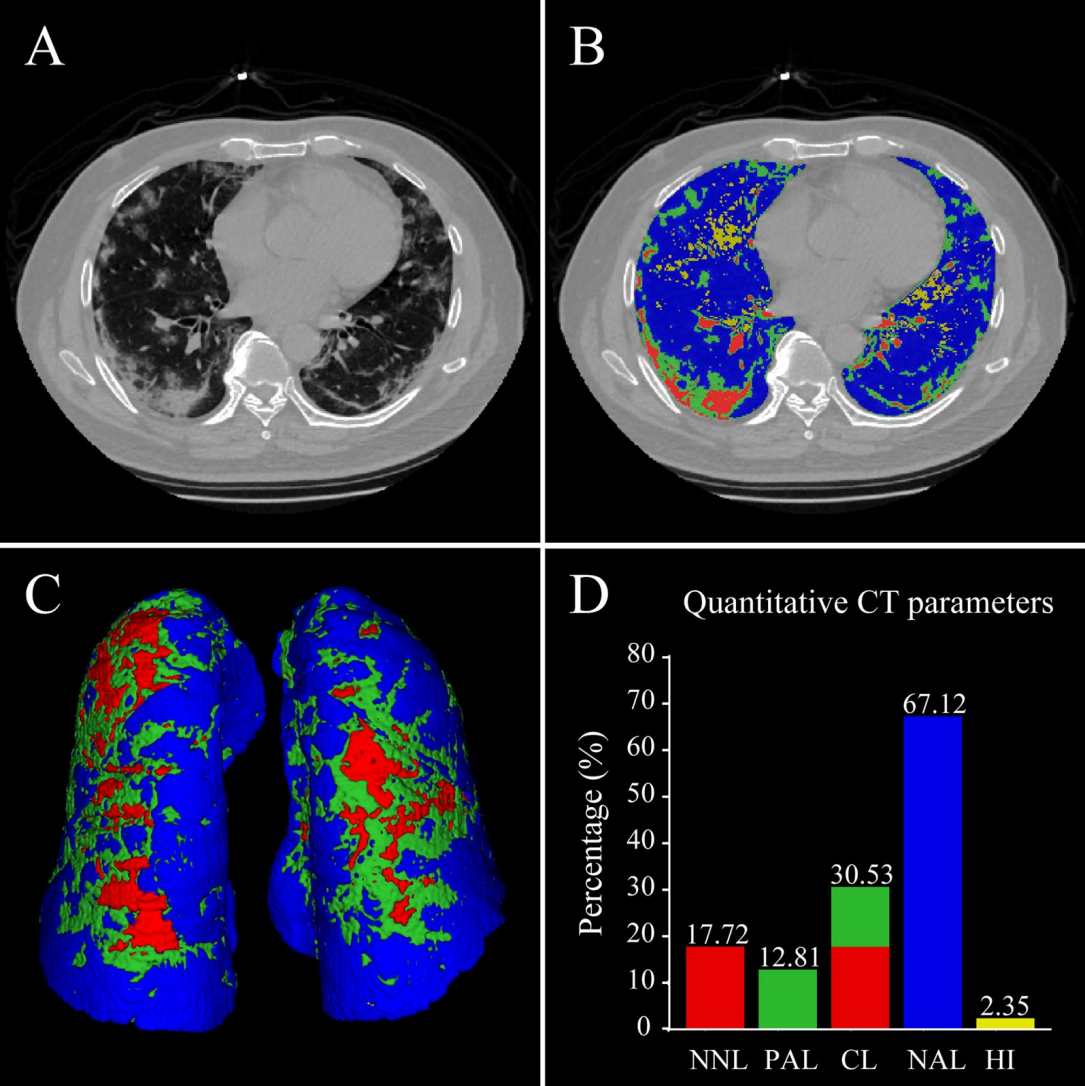
Quantitative lung CT analysis of a 55-year-old male with severe COVID-19 at admission. **A** Chest CT scan showing a bilateral mixed pattern (ground-glass opacities, interstitial thickening, and consolidation). **B** Illustration of automated lung segmentation. Yellow areas represent hyperinflated regions (%HI, − 901 to − 1000 HU); blue areas indicate normally aerated regions (%NAL, − 501 to − 900 HU); green areas represent poorly aerated regions (%PAL, − 101 to − 500 HU); red areas indicate nonaerated regions (%NNL, 100 to − 100 HU). The compromised lung volume was calculated as the sum of NNL and PAL. **C** 3D volumetric representation of the bilateral lungs. **D** Comparison among different quantitative CT parameters. The patient had 30.53% of compromised lung volume (sum of 17.72% NNL and 12.81% PAL), 67.12% NAL, and 2.35% HI

**Fig. 3:**
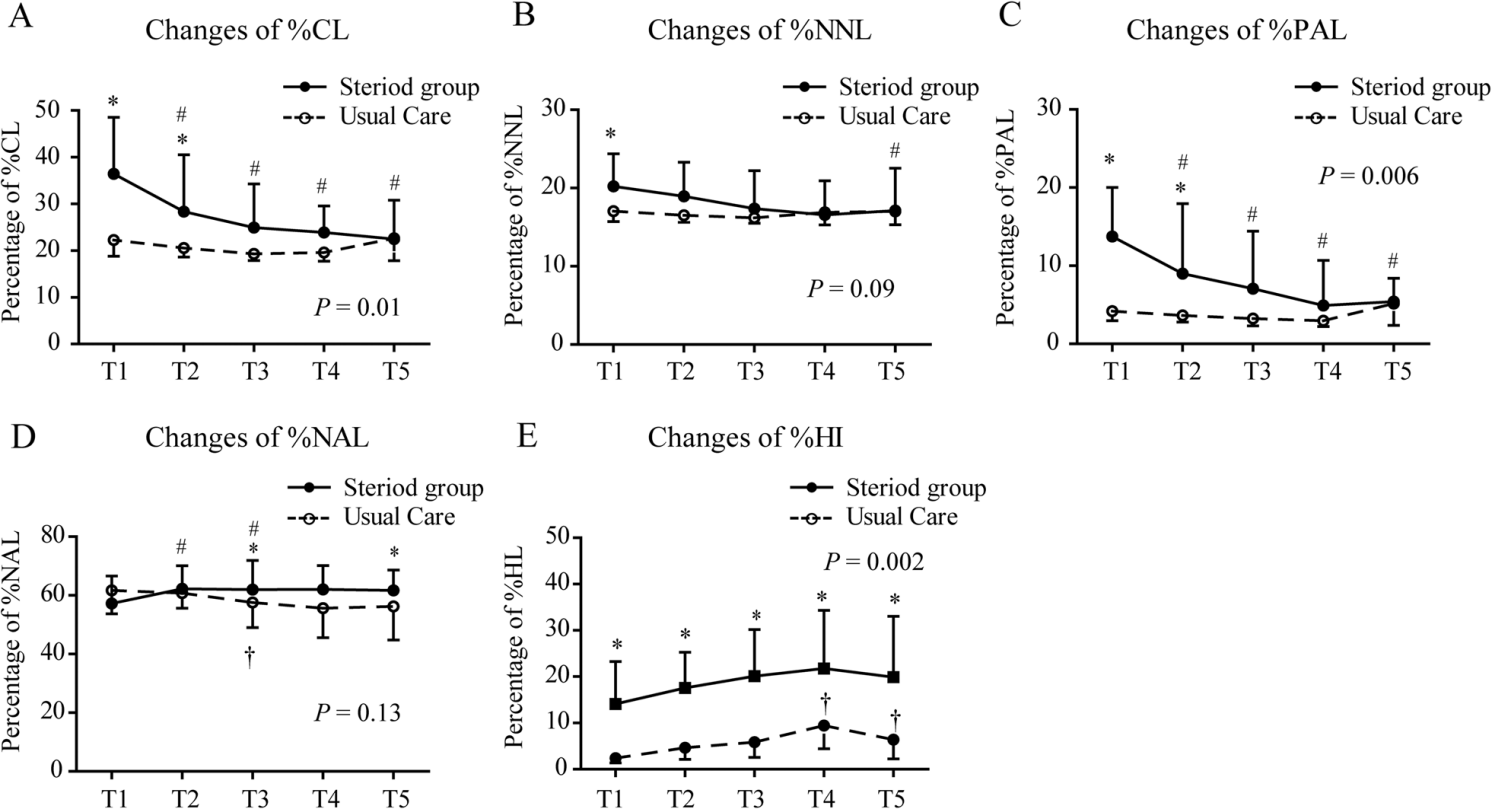
Quantitative CT parameters over time in the steroid therapy group and the standard care group. **A** %CL, compromised lung volume, considered as the sum of %PAL and %NNL; *P* < 0.001 for change over time; *P* = 0.01 for between-group difference. **B** %NNL, percentage of nonaerated lung volume; *P* = 0.08 for change over time; *P* = 0.09 for between-group difference. **C** %PAL, percentage of poorly aerated lung volume; *P* < 0.001 for change over time; *P* = 0.006 for between-group difference. **D** %NAL, percentage of normally aerated lung volume;* P* = 0.04 for change over time; *P* = 0.13 for between-group difference. **E** %HI, percentage of hyperinflated lung volume; *P* < 0.001 for change over time; *P* = 0.002 for between-group difference. *P* values for between-group difference were calculated by repeated-measures analysis of variance (ANOVA). The trends over time in quantitative CT parameters were also assessed using repeated-measures ANOVA. SIDAK multiple comparisons correction was used to compare each stage against the baseline (T1). * denotes a significant difference between two groups at each stage. # denotes a significant difference in the steroid therapy group when comparing each stage against the baseline (T1). † denotes a significant difference in the standard care group when comparing each stage against the baseline (T1). All CT scans were classified into five stages according to the interval between hospital admission and follow-up CT scans: Stage 1 (T1, at admission); Stage 2 (T2, 3–7 days); Stage 3 (T3, 8–14 days); Stage 4 (T4, 15–21 days); and Stage 5 (T5, 22–31 days)

**Fig. 4:**
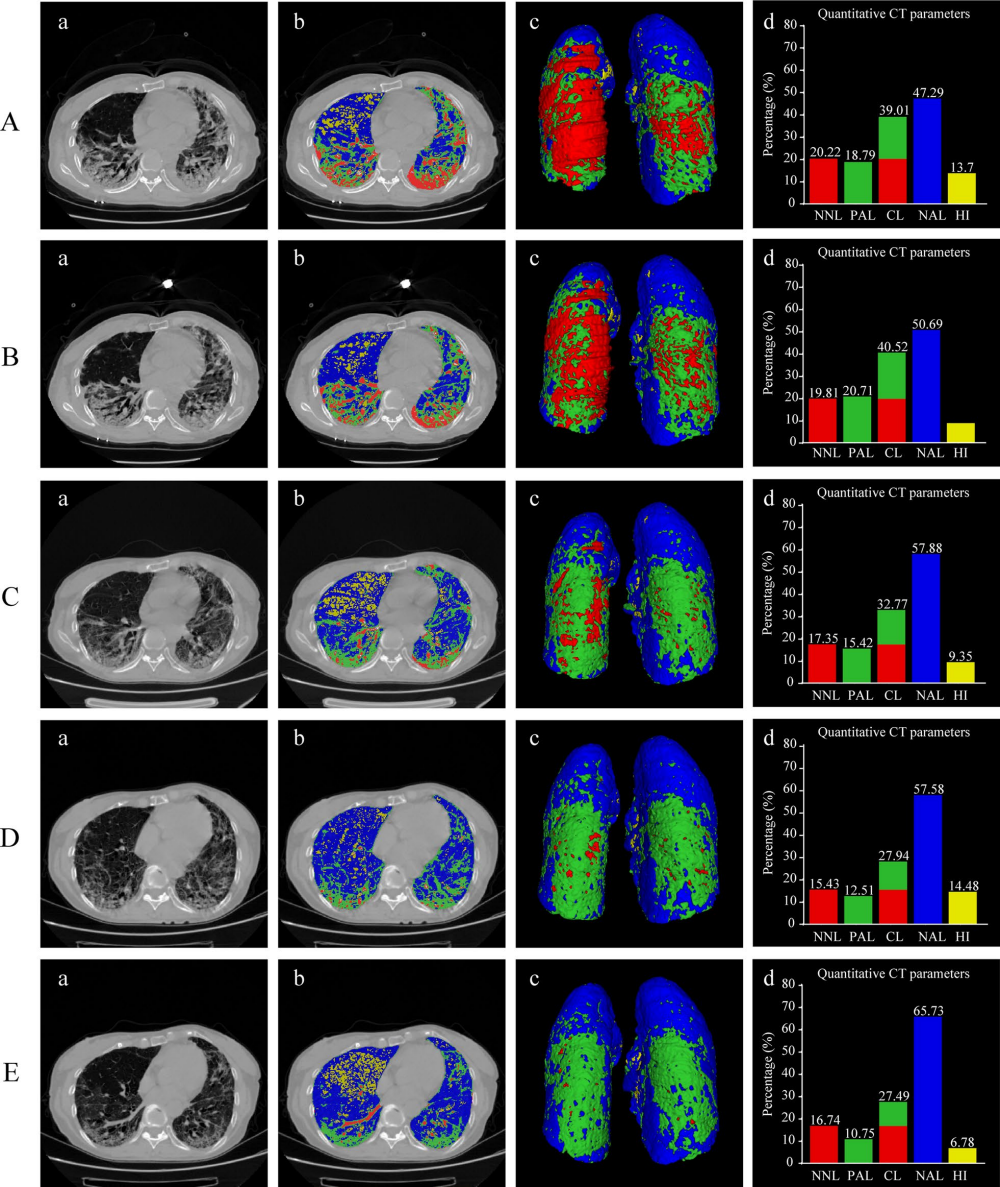
Example of quantitative lung CT analysis for a patient with severe COVID-19 receiving steroid therapy. A 69-year-old female complained of fever for 19 days accompanied with dyspnea and fatigue. After admission to hospital, she received high-flow nasal cannula oxygen therapy, arbidol, and steroid therapy. At admission, intravenous methylprednisolone was initiated with 40 mg every 12 h (1.31 mg/kg/d) for 5 days, followed by gradual tapering by 0.5 mg/kg every 5 days. Methylprednisolone was withdrawn at hospital day 15. Chest CT scans were performed at admission (**A**), day 3 (**B**), day 9 (**C**), day 16 (**D**), and day 22 (**E**). Chest CT scans (**a**), illustration of automated lung segmentation (**b**), 3D volumetric representation of the bilateral lungs (**c**), and comparison among different quantitative CT parameters (**d**) are shown in Fig. 3 at each stage. Yellow areas represent hyperinflated regions (%HI, − 901 to − 1000 HU); blue areas indicate normally aerated regions (%NAL, − 501 to − 900 HU); green areas represent poorly aerated regions (%PAL, − 101 to − 500 HU); red areas indicate nonaerated regions (%NNL, 100 to − 100 HU). The compromised lung volume was calculated as the sum of NNL and PAL. During the treatment process, the compromised lung volume decreased significantly over time from 39.01% (A-d) at admission to 27.49% (E-d) at stage 5

**Fig. 5:**
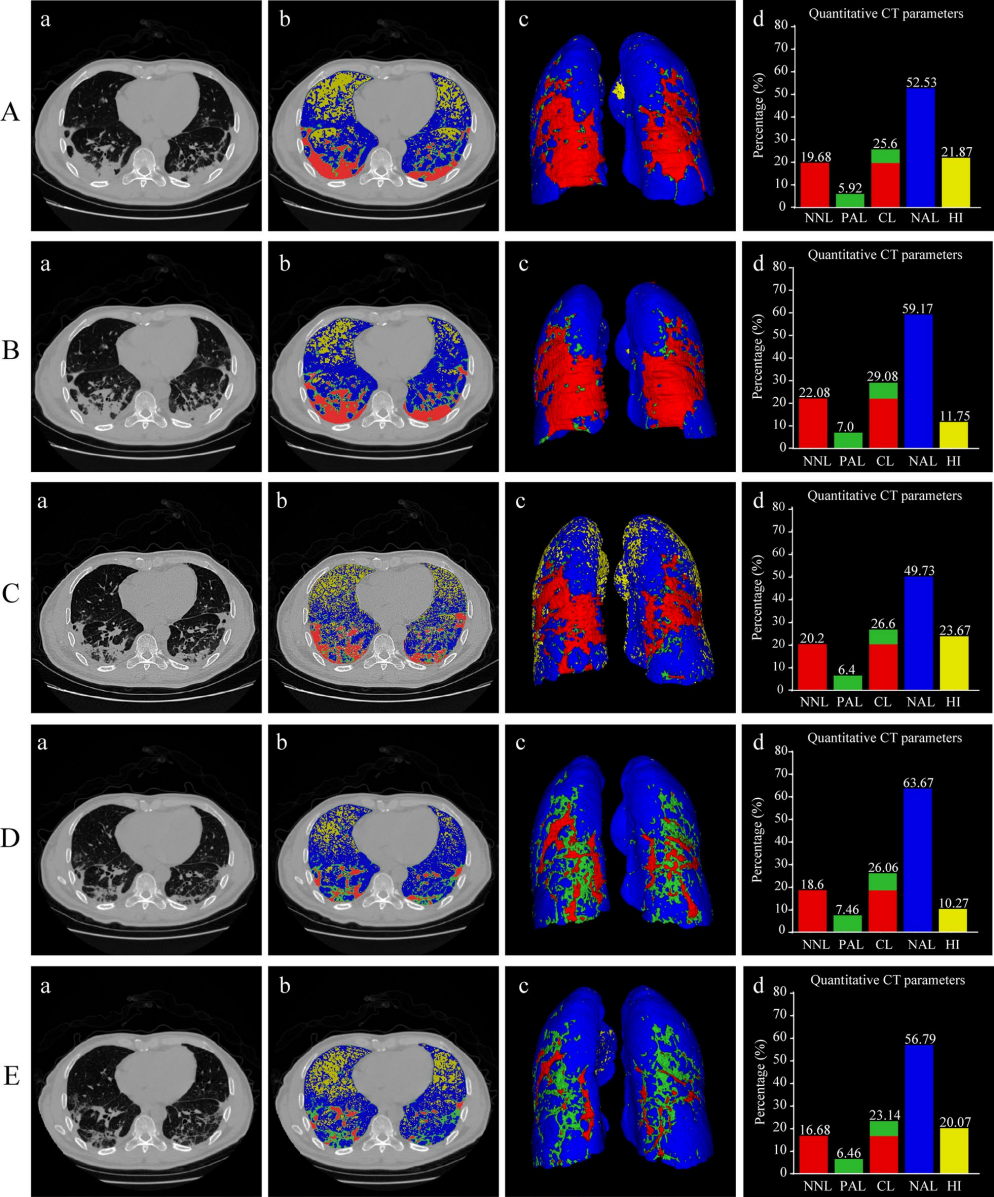
Example of quantitative lung CT analysis for a patient with severe COVID-19 with no steroid therapy. A 57-year-old male complained of fever for 12 days accompanied with dyspnea. After admission to hospital, he received high-flow nasal cannula oxygen therapy and arbidol, but without steroid therapy**.** Chest CT scans were performed at admission (**A**), day 5 (**B**), day 13 (**C**), day 19 (**D**), and day 21 (**E**). Chest CT scans (**a**), illustration of automated lung segmentation (**b**), 3D volumetric representation of the bilateral lungs (**c**), and comparison among different quantitative CT parameters (**d**) are shown in Fig. 4 at each time point. Yellow areas represent hyperinflated regions (%HI, − 901 to − 1000 HU); blue areas indicate normally aerated regions (%NAL, − 501 to − 900 HU); green areas represent poorly aerated regions (%PAL, − 101 to − 500 HU); red areas indicate nonaerated regions (%NNL, 100 to − 100 HU). The compromised lung volume was calculated as the sum of NNL and PAL. During the treatment process, the compromised lung volume showed no significant change over time from 25.6% (**A**-**d**) at admission to 23.14% (**E**-**d**) at stage 5

The quantitative percentages and volumes of compromised lung (CL), nonaerated lung (NNL), poorly aerated lung (PAL), normally aerated lung (NAL), and hyperinflated lung (HI) were extracted from CT scans according to the recommended protocols (Fig. 2 and Table 2). Quantitative CT parameters over time in the steroid therapy group and the standard care group are shown in Fig. 3. At baseline (Stage 1), the percentages of CL, NNL, PAL, and HI were higher in the steroid therapy group than in the standard care group (all *P* < 0.05; Table 2). The percentages of %CL, %PAL, and %HI were significantly decreased in the steroid therapy group as compared to those in the standard care group during the follow-up period (all *P* < 0.05 by repeated-measures ANOVA; Fig. 3A, C, E), whereas the percentage of %NNL and %NAL did not significantly differ between the groups (*P* > 0.05 by repeated-measures ANOVA; Fig. 3B, D). Examples of quantitative lung CT analysis for patients with severe COVID-19 receiving steroid therapy and with no steroid therapy were presented in Figs. 4 and 5, respectively.

**Table 2:** Quantitative chest CT parameters (%) during the follow-up period

Quantitative chest CT parameters (%)	Stage 1			Stage 2			Stage 3		
	Entire cohort (n = 72)	Steroid group (n = 31)	Standard care group (n = 41)	Entire cohort (n = 62)	Steroid group (n = 27)	Standard care group (n = 35)	Entire cohort (n = 65)	Steroid group (n = 29)	Standard care group (n = 36)
%CL	23.90 [19.83,36.11]	36.45 [25.12,48.53]*	22.29 [18.81,24.36]	22.38 [19.89,29.54]	28.35 [22.47,40.52]*	20.57 [18.63,24.13]	21.92 [18.88,31.81]	24.95 [20.90,34.29]*	19.30 [17.91,26.73]
%NNL	18.02 [15.91,21.35]	20.22 [17.72,24.37]*	17.05 [15.71,18.53]	17.26 [16.01,20.15]	18.93 [17.19,23.28]*	16.52 [15.60,18.03]	17.03 [15.62,20.75]	17.35 [16.35,22.23]	16.20 [15.50,20.13]
%PAL	5.92 [3.81,13.59]	13.73 [7.06,20.00]*	4.18 [2.97,5.97]	5.10 [3.47,8.74]	8.98 [5.88,17.93]*	3.63 [2.78,5.67]	4.67 [2.87,9.79]	7.05 [4.57,14.42]*	3.24 [2.31,6.38]
%NAL	59.23 [50.79,67.26]	57.20 [46.68,66.59]	61.69 [53.69,68.51]	61.31 [55.10,70.01]	62.25 [53.28,70.09]	60.78 [55.65,69.75]	58.72 [51.95,67.24]	61.96 [54.71,71.88]*	57.59 [49.00,62.34]
%HI	7.62 [2.12,19.40]	2.39 [1.40,7.72]*	14.11 [6.90,23.29]	7.93 [3.44,19.27]	4.65 [2.13,7.23]*	17.58 [7.17,25.27]	10.57 [3.50,22.23]	5.87 [2.57,11.79]*	20.12 [5.72,30.17]

Quantitative chest CT parameters (%)	Stage 4			Stage 5				
	Entire cohort (n = 56)	Steroid group (n = 29)	Standard care group (n = 27)	Entire cohort (n = 45)	Steroid group (n = 25)	Standard care group (n = 20)		
%CL	21.44 [18.18,27.84]	23.91 [19.80,29.57]*	19.61 [17.73,26.50]	22.49 [18.44,30.80]	22.49 [18.56,30.80]	22.65 [17.85,30.37]		
%NNL	16.72 [15.52,19.40]	16.56 [15.78,20.92]	16.88 [15.28,18.94]	17.10 [15.35,22.47]	17.10 [15.40,22.53]	17.00 [15.31,22.08]		
%PAL	4.28 [2.63,8.49]	4.92 [3.83,10.69]*	2.97 [2.20,7.46]	5.41 [2.66,8.15]	5.41 [2.95,8.38]	5.16 [2.37,7.12]		
%NAL	58.46 [53.41,68.98]	62.01 [55.39,70.18]*	55.64 [45.57,62.51]	60.32 [53.37,66.56]	61.72 [56.39,68.62]*	56.25 [44.75,62.06]		
%HI	12.81 [5.64,25.04]	9.42 [4.46,14.46]*	21.78 [8.42,34.32]	13.21 [3.45,23.48]	6.38 [2.22,17.04] **	19.90 [4.28,33.03]		

Data are expressed as the median with interquartile range in square brackets for non-normally distributed data. The Kruskal–Wallis analysis of variance was used for non-normally distributed data comparison

%CL, percentage of compromised lung volume, considered as the sum of %PAL and %NNL; %NNL, percentage of nonaerated lung volume; %PAL, percentage of poorly aerated lung volume; %NAL, percentage of normally aerated lung volume; %HI, percentage of hyperinflated lung volume.* denotes *P* < 0.05 between two groups at each stage

## Associations between steroid administration and changes in quantitative chest CT parameters during the follow-up period

### Main effect associations

GEE models were used to assess the effect of steroid on changes in quantitative chest CT parameters during the follow-up period. The main effect analyses, without interactions in the GEE model, are shown in Table 3. Across the entire cohort, univariate GEE analysis revealed that steroid administration was associated with decrease in %CL (− 7.44% [95% CI, − 10.01 to − 4.87, *P* < 0.001]) and increase in %NAL (7.46% [95% CI, 4.03 to 10.88, *P* < 0.001]). After adjusting for duration and baseline quantitative CT parameters (%), steroid administration was still associated with decrease in %CL (− 3.27% [95% CI, − 5.86 to − 0.68, *P* = 0.01]) and increase in %NAL (6.17% [95% CI, 3.51 to 8.83, *P* < 0.001]). There were no significant associations between steroid administration and changes in %NNL, %PAL, and %HI (Table 3, all *P* > 0.1).

**Table 3:** Associations between steroid administration and changes in quantitative chest CT parameters (%) in the entire cohort

Outcome	Unadjusted			Adjusted		
Changes in quantitative CT parameters (%)	Coefficient	95% CI	*P* value	Coefficient	95% CI	*P* value
Changes in CL^a^	− 7.44	− 10.01 to − 4.87	< 0.01	− 3.27	− 5.86 to − 0.68	0.01
Changes in NNL^b^	− 1.75	− 3.01 to − 0.48	< 0.01	− 0.68	− 1.61 to 0.24	0.15
Changes in PAL^c^	− 5.71	− 7.66 to − 3.77	< 0.01	− 1.44	− 3.46 to 0.57	0.16
Changes in NAL^d^	7.46	4.03 to 10.88	< 0.01	6.17	3.51 to 8.83	< 0.01
Changes in HI^e^	0.06	− 2.81 to 2.92	0.97	− 1.86	− 4.73 to 1.01	0.20

Analyses using generalized estimating equations to implement linear regression models. Coefficients represent differences in changes in quantitative chest CT parameters associated with steroid administration. All analyses were adjusted for duration and baseline quantitative chest CT parameters. ^a^ Model adjusted for duration and baseline %CL; ^b^ Model adjusted for duration and baseline %NNL; ^c^ Model adjusted for duration and baseline %PAL; ^d^ Model adjusted for duration and baseline %NAL; ^e^ Model adjusted for duration and baseline %HI. %CL, percentage of compromised lung volume, calculated as the sum of %PAL and %NNL; %NNL, percentage of nonaerated lung volume; %PAL, percentage of poorly aerated lung volume; %NAL, percentage of normally aerated lung volume; %HI, percentage of hyperinflated lung volume

### Interaction by duration

Statistically significant interactions were observed between steroid administration and duration for quantitative chest CT parameters (all interactions, *P* < 0.05). Therefore, we analyzed the association between steroid administration and change in quantitative chest CT parameters at each stage separately (Table 4). At stage 2, steroid administration was not associated with changes in %CL (− 2.02%, [95% CI, − 4.55 to 0.51], *P* = 0.12). However, after stage 3, steroid administration was significantly associated with decreases in %CL. Steroid administration was associated with a 3.73% decrease in %CL ( [95% CI, − 7.18 to − 0.29], *P* = 0.03) at stage 3, a 3.95% decrease in %CL ( [95% CI, − 7.57 to − 0.33], *P* = 0.03) at stage 4, and a 5.01% decrease in %CL ( [95% CI, − 8.95 to − 1.08], *P* = 0.01) at stage 5. Steroid administration at each stage was significantly associated with changes in %NAL. Steroid administration was associated with a 3.65% increase in %NAL ( [95% CI, 0.68 to 6.63], *P* = 0.02) at stage 2, a 8.23% increase in %NAL ( [95% CI, 4.50 to 11.96], *P* = 0.000) at stage 3, a 7.82% increase in %NAL ( [95% CI, − 3.41 to 12.23], *P* = 0.001) at stage 4, and a 9.59% increase in %NAL ( [95% CI, 4.65 to 14.53], *P* = 0.000) at stage 5. No associations were observed between steroid administration and changes in %NNL or %HI after analyzing each stage separately. Steroid administration was also not associated with changes in %PAL at all stages, except stage 5.

**Table 4:** Associations between steroid administration and changes in quantitative CT parameters (%) at different stages

Outcome	Stage 2			Stage 3			Stage 4			Stage 5		
Changes in quantitative CT parameters (%)	Coefficient	95% CI	*P* value	Coefficient	95% CI	*P* value	Coefficient	95% CI	*P* value	Coefficient	95% CI	*P* value
Changes in CL ^a^	− 2.02	− 4.55 to 0.51	0.12	− 3.73	− 7.18 to − 0.29	0.03	− 3.95	− 7.57 to − 0.33	0.03	− 5.01	− 8.95 to − 1.08	0.01
Changes in NNL ^b^	− 0.23	− 1.49 to 1.02	0.71	− 0.83	− 2.34 to 0.68	0.28	− 1.15	− 2.99 to 0.69	0.22	− 1.07	− 3.06 to 0.92	0.28
Changes in PAL ^c^	− 1.01	− 2.74 to 0.72	0.25	− 2.31	− 4.71 to 0.08	0.06	− 1.85	− 4.10 to 0.40	0.11	− 2.99	− 5.24 to − 0.75	0.01
Changes in NAL ^d^	3.65	0.68 to 6.63	0.02	8.23	4.50 to 11.96	< 0.01	7.82	3.41 to 12.23	< 0.01	9.59	4.65 to 14.53	< 0.01
Changes in HI ^e^	− 2.54	− 5.96 to 0.88	0.14	− 2.73	− 6.91 to 1.45	0.20	− 1.77	− 7.01 to 3.47	0.50	− 3.27	− 8.67to 2.12	0.23

Analyses were performed using linear regression models adjusted for baseline quantitative chest CT parameters. Coefficients represent differences in changes in quantitative CT parameters associated with steroid administration. ^a^ Model adjusted for duration and baseline %CL; ^b^ Model adjusted for duration and baseline %NNL; ^c^ Model adjusted for duration and baseline %PAL; ^d^ Model adjusted for duration and baseline %NAL; ^e^ Model adjusted for duration and baseline %HI. %CL, percentage of compromised lung volume, calculated as the sum of %PAL and %NNL; %NNL, percentage of nonaerated lung volume; %PAL, percentage of poorly aerated lung volume; %NAL, percentage of normally aerated lung volume; %HI, percentage of hyperinflated lung volume

### Interaction by disease severity

Associations between steroid administration and changes in %CL, %NNL, %PAL, %NAL, or %HI during the follow-up period varied between different baseline quantitative CT parameters (all interactions, *P* < 0.01). All patients were classified into high (> median level) and low groups (< median level) according to baseline quantitative CT parameters. On the basis of the analysis of different quantitative parameters separately, the associations between steroid administration and changes in quantitative CT parameters are shown in Table 5. Steroid administration was associated with more significant decrease in %CL in the high CL group (-9.67%, [95% CI, − 13.12 to − 6.22], *P* < 0.001), but there was no significant association between steroid administration and %CL decrease in the low CL group (0.81%, [95% CI, − 1.72 to 3.34], *P* = 0.53). Similarly, steroid administration was associated with a greater decrease in NNL or PAL in the high NNL or PAL group, but the association was not significantly different in the low NNL or PAL group. For NAL, steroid administration was associated with significant increases in %NAL in both high NAL group (5.92%, [95% CI, 1.88 to 9.95], *P* = 0.004) and low NAL group (7.28%, [95% CI, 2.59 to 11.97], *P* = 0.002). Steroid administration was, however, not associated with changes in HI in both high and low HI groups (all *P* > 0.1).

**Table 5:** Associations between steroid administration and changes in quantitative chest CT parameters (%) according to baseline parameters

Outcome	Coefficient	95% CI	*P* value
Changes in CL (%)			
High CL group (≥ 23.90)	− 9.67	− 13.12 to − 6.22	< 0.01
Low CL group (< 23.90)	0.81	− 1.72 to 3.34	0.53
Changes in NNL (%)			
High NNL group (≥ 18.02)	− 1.88	− 3.63 to − 0.12	0.04
Low NNL group (< 18.02)	− 0.17	− 1.20 to 0.86	0.75
Changes in PAL (%)			
High PAL group (≥ 5.92)	− 6.74	− 9.29 to − 4.19	< 0.01
Low PAL group (< 5.92)	0.96	− 1.00 to 2.92	0.34
Changes in NAL (%)			
High NAL group (≥ 59.23)	5.92	1.88 to 9.95	< 0.01
Low NAL group (< 59.23)	7.28	2.59 to 11.97	< 0.01
Changes in HI (%)			
High HI group (≥ 7.62)	− 1.84	− 8.12 to 4.44	0.57
Low HI group (< 7.62)	− 0.35	− 3.73 to 3.02	0.84

Analyses using generalized estimating equations to implement linear regression models. Coefficients represent the differences in changes in quantitative CT parameters associated with steroid administration. All analyses were adjusted for duration. %CL, percentage of compromised lung volume, calculated as the sum of %PAL and %NNL; %NNL, percentage of nonaerated lung volume; %PAL, percentage of poorly aerated lung volume; %NAL, percentage of normally aerated lung volume; %HI, percentage of hyperinflated lung volume

## Discussion

To the best of our knowledge, the present study is the first to assess the association between steroid administration and quantitative CT variables in patients with COVID-19. Our results showed that steroid administration was independently associated with decreases in compromised lung volume (%CL, − 500 to 100 HU) with a significant interaction by duration or disease severity in a longitudinal cohort.

Chest CT plays an important role in differentiating, diagnosing, and monitoring pulmonary disease progression [13]. Chest CT has been included as an important modality for COVID-19 diagnosis and management in the sixth edition of Chinese national guidelines on the diagnosis and treatment for COVID-19 [16]. Several QCT analysis approaches have been developed recently for quantifying a range of lesions. The visual or semi-quantitative analysis methods (i.e., “CT severity score”) were used for assessing disease severity [26, 27]. However, because of large inter- and intra-observer variability, visual or semi-quantitative assessment of CT findings cannot accurately and quantitatively monitor disease progression and treatment response [9]. In our preliminary study, we found that quantitative parameters of QCT may serve as a useful endpoint in evaluating treatment efficacy in patients with COVID-19 [13]. Further, we found that patients with severe COVID-19 receiving steroid therapy showed a significant different recovery pattern compared to those not receiving steroid therapy using a deep learning method [14]. However, due to the black-box brought by deep networks, we were not able to further interpret the difference we have observed. In the present study, we performed a computer-aided quantitative lung lesion extraction analysis to assess lung lesions. The software (3D-slicer) we used for lung segmentation has been specifically updated recently to support the urgent need of segmenting compromised lung parenchyma in COVID-19 pandemic (https://github.com/rbumm/SlicerLungCTAnalyzer/). In the present study, threshold-based quantitative analyses were conducted for the aerated-condition segmentation process, which provided standardized and reproducible evaluation for lung lesions [12]. This method has been confirmed to predict the need for oxygenation support and intubation in patients with COVID-19 [25]. Compromised lung volume, including NNL and PAL, is significantly correlated with respiratory dysfunction and can accurately predict poor outcomes in patients with COVID-19 [25, 28]. Hence, we chose changes in percentage of compromised lung volume (Δ%CL) at different stages as the primary outcome to evaluate response to steroid therapy.

In this study, the steroid therapy group exhibited a higher baseline compromised lung volume and longer hospital stay than the standard care group, although vital signs and the PaO_2_/FiO_2_ ratio at the start of therapy were comparable in both groups. This indicated that steroids were used in patients who were relatively critically ill. Patients receiving steroid therapy showed a significant decrease in compromised lung volume as compared to those without steroid therapy, which indicates a faster resolution rate in the steroid therapy group.

The association between steroid administration and decrease in %CL varied between different stages. In stage 2 (within 1 week of admission), steroid therapy did not show an association with decrease in %CL. However, after 1 week of admission (stages 3–5), steroid therapy showed a significant association with decrease in %CL. Similarly, steroid administration at each stage was significantly associated with changes in %NAL. Steroid administration was associated with a small increase in %NAL at stage 2. At stage 3–5, steroid therapy showed a significant association with increase in %NAL. These indicated that steroid therapy exerted an effect of long-term radiographic improvement, rather than an early short-term effect. These findings were consistent with a previous study, in which steroid therapy was found to decrease late treatment failure in patients with severe community-acquired pneumonia and high inflammatory response, primarily due to decrease in radiographic progression [29].

Patients with severe COVID-19 usually rapidly progress to acute respiratory distress syndrome (ARDS) at an early stage, which is mainly related to dysregulated immune response [30–32]. Theoretically, with several anti-inflammatory and immunomodulatory properties, corticosteroids could prevent an excessive immune response and might also prevent the progression of COVID-19 [33, 34]. Pooled results from recent RCTs in patients with severe or critical COVID-19 showed a significant reduction in mortality following the use of systemic corticosteroids [3, 35, 36]. However, the use of corticosteroid therapy is highly controversial for patients with severe pneumonia, including SARS, Middle East respiratory syndrome (MERS), influenza, and community-acquired pneumonia [37–40]. It can be inferred that the discrepancy may be attributed to heterogeneity in the type of corticosteroids administered, timing of therapy initiation, dose, medical conditions, and disease severity.

In the present study, the association between steroid administration and decreases in %CL varied with disease severity. Steroid therapy was associated with a decrease in %CL in the high CL group, but not in the low CL group. For %NAL, steroid administration was associated with significant increase in %NAL in both high NAL group and low NAL group. But the β coefficient in high NAL group was higher than that in low NAL group. These indicated that steroid therapy had a greater effect in patients with high disease severity. The findings were supported by recent studies [2, 41, 42], in which corticosteroid therapy led to different clinical outcomes according to severity of illness. We speculated that the absence of benefit in the low CL group may be partially explained by the delayed viral clearance [43–45]. In the present study, patients in the low CL group receiving steroids had relatively longer viral shedding duration than those who were not receiving steroids, although the difference was not statistically significant. Although recent WHO guidelines recommended steroid therapy for patients with severe COVID-19 [15], our study showed that steroid therapy may not be appropriate for all those patients. Further studies may be needed to clarify the optimal indication of steroid therapy.

The present study has several limitations. First, the small sample size in this longitudinal study and missing CT scans during the treatment process might restrict the statistical power. Second, our findings cannot be generalized to all patients with COVID-19 as most critical patients with less than two serial CT scans were excluded from this cohort. Moreover, selective biases in the type of corticosteroids administered, dose, duration, and dose tapering might affect the efficacy of corticosteroids in patients with severe COVID-19. Third, adverse outcomes related to steroid therapy, such as opportunistic infections, hyperglycemia, and neuromyopathy, were not assessed in this study. Fourth, as arteries and bronchi were not excluded from lung segmentation, these interstitial structures may partially fall in the same threshold as the nonaerated lung, which might make the quantification of compromised lung volume inaccurate. All patients with COVID-19 in our study had severe infection, and therefore, the effect of additional volume for lung interstitial structure was very limited. However, because the same method was used for all patients, the potential inaccuracy in the estimation of compromised lung volume was counterbalanced to some extent.

## Conclusions

Steroid administration was independently associated with decrease in compromised lung volume (%CL, − 500 to 100 HU) with significant interaction by duration or disease severity in a longitudinal cohort. The QCT parameters, particularly compromised lung volume, may provide clinicians with an accurate tool to assess or monitor the progression of COVID-19 during the treatment process. Future large-scale prospective studies are warranted to validate our findings.

## Data Availability

The datasets used and/or analyzed during the current study are available from the corresponding author on reasonable request.

## Abbreviations

CL: Compromised lung
CI: Confidence interval
COVID-19: Coronavirus disease 2019
FiO_2_: Fraction of inspiration oxygen
GEE: Generalized estimating equations
HI: Hyperinflated lung
HU: Hounsfield unit
IQR: Interquartile range
NAL: Normally aerated lung
NNL: Nonaerated lung
PAL: Poorly aerated lung
PaO_2_: Partial arterial oxygen pressure
QCT: Quantitative computed tomography
SD: Standard deviation
